# Th1 Response and Systemic Treg Deficiency in Inclusion Body Myositis

**DOI:** 10.1371/journal.pone.0088788

**Published:** 2014-03-04

**Authors:** Yves Allenbach, Wahiba Chaara, Michelle Rosenzwajg, Adrien Six, Nicolas Prevel, Federico Mingozzi, Julia Wanschitz, Lucile Musset, Jean-Luc Charuel, Bruno Eymard, Benoit Salomon, Charles Duyckaerts, Thierry Maisonobe, Odile Dubourg, Serge Herson, David Klatzmann, Olivier Benveniste

**Affiliations:** 1 Immunlogy-Immunopathology-Immunotherpapy (I3), Sorbonne Universités, Pierre and Marie Curie University Paris 06, Paris, France; 2 Immunlogy-Immunopathology-Immunotherpapy (I3), Centre National de la Recherche Scientifique UMR 7211, Paris, France; 3 Immunlogy-Immunopathology-Immunotherpapy (I3), UMRS_959, Institut National de la Santé et de la Recherche Médicale, Paris, France; 4 Inflammation-Immunopathology-Biotherapy (i2B), Hôpital Pitié-Salpêtrière, Assistance Publique - Hôpitaux de Paris, Paris, France; 5 Internal Medicine Department 1, Centre de référence Maladie Neuromusculaire, Assistance Publique - Hôpitaux de Paris, Hôpital Pitié-Salpêtrière Paris, France; 6 U974, Sorbonne Universités, Pierre and Marie Curie University, Paris 06, Paris, France; 7 U974, Institut National de la Santé et de la Recherche Médicale, Paris, France; 8 Genethon, Evry, France; 9 Department of Neurology, Innsbruck Medical University, Innsbruck, Austria; 10 Department of immunochemistry, Hôpital Pitié-Salpêtrière, Assistance Publique - Hôpitaux de Paris, Pierre and Marie Curie University Paris 06, Paris, France; 11 Department of neurology, Hôpital Pitié-Salpêtrière, Assistance Publique - Hôpitaux de Paris, Pierre and Marie Curie University, Paris 06, Paris, France; 12 Department of neuropathology, Hôpital Pitié-Salpêtrière, Assistance Publique - Hôpitaux de Paris, Pierre and Marie Curie University Paris 06, Paris, France; University of São Paulo, Brazil

## Abstract

**Objective:**

Sporadic inclusion body myositis (sIBM), the most frequent myositis in elderly patients, is characterized by the presence muscle inflammation and degeneration. We aimed at characterizing immune responses and regulatory T cells, considered key players in the maintenance of peripheral immune tolerance, in sIBM.

**Methods:**

Serum and muscle tissue levels of 25 cytokines and phenotype of circulating immune cells were measured in 22 sIBM patients and compared with 22 healthy subjects. Cytokine data were analysed by unsupervised hierarchical clustering and principal components analysis.

**Results:**

Compared to healthy controls, sIBM patients had increased levels of Th-1 cytokines and chemokines such as IL-12 (261±138 pg/mL vs. 88±19 pg/mL; p<0.0001), CXCL-9 (186±12 pg/mL vs. 13±7 pg/mL; p<0.0001), and CXCL-10 (187±62 pg/mL vs. 13±6 pg/mL; p<0.0001). This was associated with an increased frequency of CD8^+^CD28^−^ T cells (45.6±18.5% vs. 13.5±9.9%; p<0.0001), which were more prone to produce IFN-γ (45.6±18.5% vs. 13.5±9.9%; p<0.0001). sIBM patients also had a decreased frequency of circulating regulatory T cells (CD4^+^CD25^+^CD127^low^FOXP3^+^, 6.9±1.7%; vs. 5.2±1.1%, p = 0.01), which displayed normal suppressor function and were also present in affected muscle.

**Conclusion:**

sIBM patients present systemic immune activation with Th1 polarization involving the IFN-γ pathway and CD8^+^CD28^−^ T cells associated with peripheral regulatory T cell deficiency.

## Introduction

Idiopathic inflammatory myopathies are a group of acquired muscular disorders including polymyositis (PM), dermatomyositis (DM), immune-mediated necrotizing myopathies (IMNM), and sporadic inclusion body myositis (sIBM). sIBM is a rare disease, which differs from the other idiopathic inflammatory myopathies because it affects more frequently elderly subjects. Muscle weakness progression in sIBM is generally very slow and refractory to immunosuppressants [1]. The muscle inflammatory pattern in PM and sIBM is very similar, with upregulation of class I major histocompatibility complex (MHCI) molecules on the surface of muscle fibres, and muscle infiltration by cytotoxic CD8^+^ T cells, suggesting an autoimmune responses against muscle fibers [2]. Furthermore, the histological feature of sIBM includes presence of amyloid deposits, presumably linked to degenerative process [3]. The relative pathophysiological importance of inflammatory mechanisms is controversial in the genesis of this disease [4]. Additionally, little is known about the overall activation state of the immune system in sIBM, especially for what concerns natural regulatory T cells (Tregs) that play a key role in autoimmunity.

Thus, defining the pattern of immune activation and the balance of Treg vs. T effector responses in sIBM could lead to the development of targeted immune treatments and may provide insights into the interactions of immune system and muscle degeneration, which characterize the disease.

We observed in sIBM patients a systemic immune activation with Th1 polarization involving IFN-γ pathway and CD8^+^CD28^−^ T cells associated with peripheral Tregs deficiency, suggesting that therapeutic approaches targeting effectors Th1 T cells and sparing or increasing Treg number and function might be of interest.

## Patients and Methods

### Patients and Samples

We studied patients with a definite diagnosis of sIBM based on pathological criteria [5], i.e. presenting inflammatory infiltrates with non-necrotic fibres infiltrated by lymphocytes, rimmed vacuoles, amyloid deposits or degeneration-related proteins evidenced by TDP43 (ProteinTech Group) and P62 (clone: 3/P62 LCK ligand, BD Bioscience) immunostaining [6]. None of the subjects had received immunosuppressive or immunomodulatory drugs during the 6 months before inclusion in the study.

Sera and peripheral blood mononuclear cells (PBMCs) of 22 sIBM patients were compared to age- and sex-matched healthy subjects (n = 22) free of inflammatory/autoimmune diseases, chronic viral infection (human immunodeficient virus, or hepatitis B and C virus), past history of cancer or active cancer, and who were not receiving any immunosuppressive or immunomodulatory drugs. Frozen muscle biopsies from a subset of sIBM patients (n = 8) were studied by immunohistochemistry. Cytokine and chemokine levels in muscle lysates from sIBM patients (n = 8) were compared to those from non-myositis controls (patients with isolated myalgia and histologically normal muscle biopsy, n = 7).

In a second set of experiments, sera of 9 sIBM patients (different from those previously included) were tested and compared to sera (sampled at diagnosis before any therapeutic intervention) from other myositis patients, which included polymyositis (PM, n = 12, among them five had anti-Jo1 antibodies), dermatomyositis (DM, n = 12), and immune-mediated necrotizing myopathy associated with anti-SRP antibodies (SRP IMNM, n = 13) based on ENMC criteria [7].

The local ethics committee Groupe Hospitalier Pitie Salpetriere approved the study protocol, all the patients were enrolled after giving their written informed consent, and it received the clinicalTrials.gov number: NCT00898989.

### Flow Cytometry

Fresh peripheral blood mononuclear cells (PBMCs) were stained with the following monoclonal antibodies (mAbs): CD3, CD4, CD16, CD8, CD19, CD20, CD45RA, CD45RO, CD56 (all from Beckman Coulter, Villepinte, France), CD4, CD25, CD28, CD62L, HLA-DR (BD Biosciences, Le Pont De Claix, France), and CCR6 (eBiosciences, Paris, France).

Intranuclear detection of FoxP3 was also performed using a commercially available kit (eBioscience). For the detection of intracellular cytokine production, PBMC were stimulated with 50 ng/mL PMA and 1 mM ionomycin in the presence of Golgi-Stop (BD Biosciences) for four hours, then stained with anti-IFN-γ-FITC (eBioscience) or anti-IL-17-Alexa Fluor 647 (e-Bioscience) as recommended by the manufacturer.

Cell acquisition and analysis by flow cytometry were performed with an FC500 cytometer (Beckman Coulter). Data were analysed with CXP software (Beckman Coulter).

### T Cell Suppression Assays

The T cell suppression assays was performed as previously described [8]. Briefly, Tregs (CD3^+^CD4^+^CD25^+^CD127^low^ cells) were FACS sorted (Aria flow cytometer, BD Biosciences) and mixed at various cell ratios with autologous effector T cells (CD3^+^CD4^+^CD25^−^ cells) in the presence of irradiated allogeneic mononuclear cells as stimulator cells. Cell proliferation was determined by incorporation of tritiated thymidine (3H-Tdr, Amersham, Buckinghamshire, UK) and measured using a β-counter (counter-WALLAC).

The results were expressed as percentage inhibition using the following formula: (1 − (experimental counts per min/control counts per min) × 100%.

### Cytokine and Chemokine Assays

Concentrations of 25 cytokines and chemokines (GM-CSF, IFNα, IFNγ, IL-1RA, IL1β, IL-2, IL-2R, IL-4, IL-5, IL-6, IL-7, IL-8, IL-10, IL-12, IL-13, IL-15, IL-17, CXCL-10, CCL-2, CXCL-9, CCL-3, CCL-4, CCL-5, TNF-α, and Eotaxin) were measured in serum, and in muscle lysates by using the Human Cytokine 25-Plex assay (Invitrogen, CergyPontoise, France) as recommended by the manufacturer. Analysis of frozen muscle biopsies was performed as previously described [9].

### Histology and Immunohistochemistry

CD4^+^, FOXP3^+^, and IL-17^+^ cells were detected in muscle biopsy samples by immunohistochemistry. Acetone-fixed, frozen sections were exposed to monoclonal antibodies against CD4 (Dako clone MT-310), FoxP3 (eBioscience, clone 236A/E7), or IL-17 (R&D systems Clone AF-317-NA) overnight at 4°C. Positive staining was revealed by peroxidase reaction (Dako real™ Detection System (K5001; Dako)). Double immunofluorescence was used to analyse FoxP3^+^ and CD4^+^ cell colocalisation. Detection of mouse monoclonal anti-FoxP3 and rabbit polyclonal anti-CD4 (Abcam®) antibodies was performed using Alexa Fluor (AF) 555 goat anti-mouse IgG (L+H) and AF 488 goat anti-rabbit IgG (L+H) secondary antibodies. For control purposes, a mouse IgG1 isotype control was included in the protocol. Stained muscle sections were analyzed with a Leica TCS-SP (UV) confocal microscope at the MSSM-Microscopy Shared Resource Facility (Pitie-Salpetriere Hospital, Paris).

### Statistical Analyses

Data are presented as mean (±SD) for continuous variables and as percentage for qualitative variables. Fisher’s exact test was used to compare qualitative variables, and non-parametric Mann–Whitney and Wilcoxon tests were used to compare continuous variables. A p-value <0.05 was considered significant. Statistical analyses were performed using GraphPad Prism version 4.0 and Instat version 3.0 for Windows (GraphPad Software, San Diego, CA, USA).

For cytokine and chemokine multivariate analyses, a first quality control check step allowed identifying variables and samples for which the number of available data was insufficient. As describe before [10], differentially-expressed cytokines were used to perform principal components analysis (PCA) and unsupervised hierarchical clustering on normalised patient data. Based on the statistically significant cytokine vector (i.e. signature), a model using Linear Discriminant Analysis was built to predict the membership of a test sample to the patient or control group. For all data-processing steps and statistical and multivariate analyses, we used R software (http://www.r-project.org/).

## Results

### Analysis of Cytokines and Chemokines Levels in sIBM Patients Compared to Healthy Subjects Shows a Th1 Polarization of Immune System

In order to characterise immune responses in sIBM patients, cytokines and chemokines levels were measured in serum from 22 sIBM patients (67.9±10.3 years; 9 males, 13 females). Patients’ characteristics are reported in Table S1.

**Figure 1:**
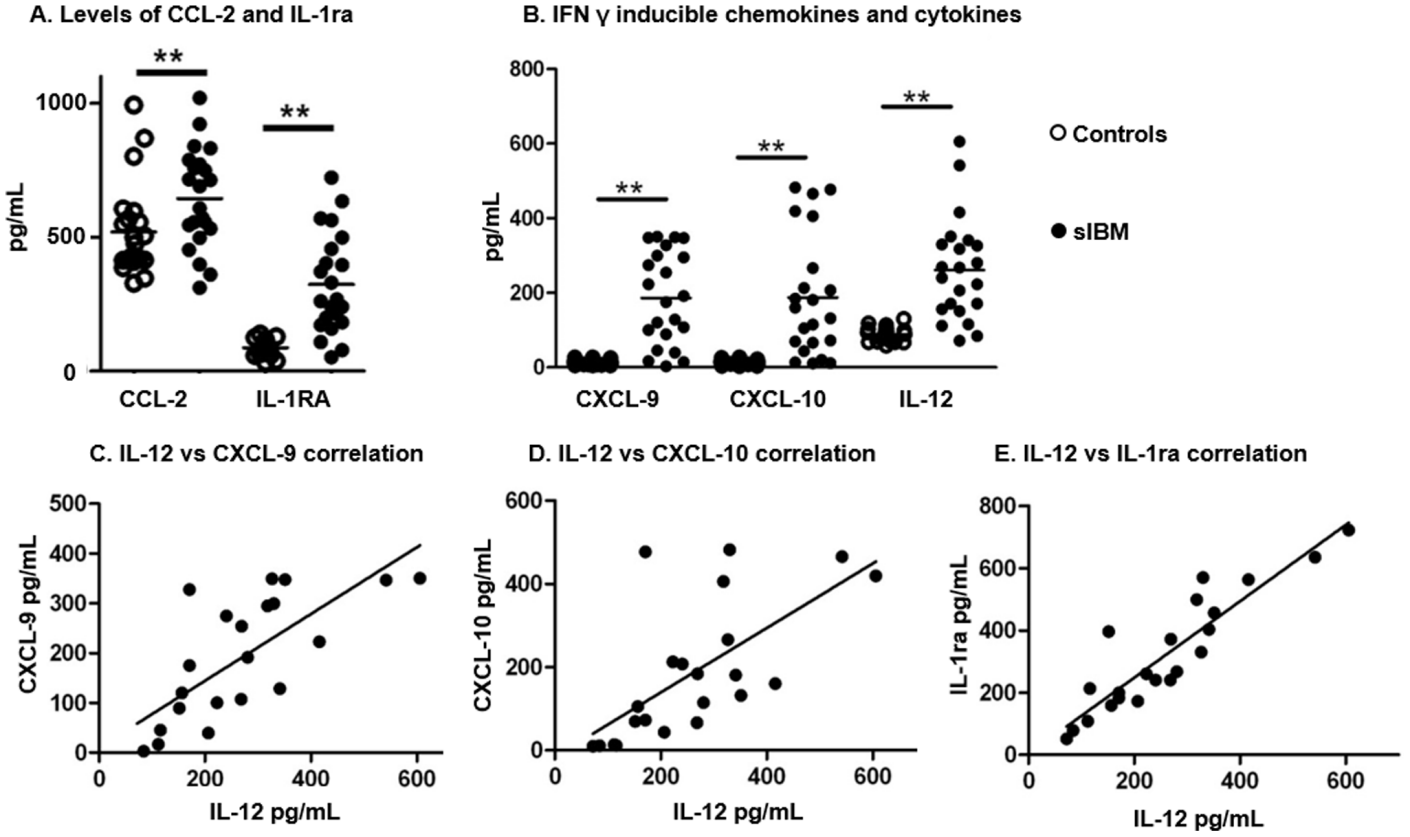
Cytokine and chemokine levels in sIBM patients compare to healthy controls. **A.** Pooled data showing serum levels (pg/mL) of CCL-2 and IL-1ra in sIBM patients and controls; p<0.0001 and p = 0.025 respectively. **B.** Pooled data showing serum levels (pg/mL) of CXCL-9, CXCL-10 and IL-12 in sIBM patients (open circles) and controls (full circles); p<0.0001 for all. **C.** Correlation (linear regression) between serum level of IL-12 and serum levels of CXCL-9 (C), CXCL-10 (**D**) and IL-1ra (**E**). Horizontal lines indicate means; *p<0.05; **p<0.001.

IL-1RA (323±187 *vs.* 87±28 pg/mL; p<0.0001) and CCL-2 (649±149 *vs.* 452±170 pg/mL; p = 0.025) levels were higher in sIBM patients than in controls (Figure 1. A), indicating the presence of systemic inflammation.

Higher levels of the Th1 chemokines CXCL-9 (186±12 pg/mL *vs.*13±7 pg/mL; p<0.0001) and CXCL-10 (187±62 pg/mL *vs.* 13±6 pg/mL; p<0.0001) were found in sIBM patients compared to controls (Figure 1. B), which was in agreement with the detection of higher levels of IL-12 in sIBM patients (261±138 pg/mL *vs.* 88±19 pg/mL; p<0.0001), a Th1 cytokine. Furthermore, a Th-1 cytokine serum levels (IL-12) correlated strongly with those of Th-1 chemokines CXCL-9 (r^2^ = 0.87; p<0.001) and CXCL-10 (r^2^ = 0.87; p<0.001, Figure 1. C–D). IL-12 levels also correlated with IL-1RA levels (r^2^ = 0.92; p<0.001, Figure 1. E).

Conversely, we observed no difference in the levels of Th2 cytokines (IL-4, IL-5, IL-10 and IL-13), either in serum or after *ex vivo* stimulation of PBMCs (data not shown), or Th-17 signature markers. The serum levels of IL-17 and the percentage of circulating Th-17 cells (CD4^+^CCR6^+^IL-17^+^ T cells) were similar in both sIBM and controls, and few IL-17^+^ cells were detected in muscle infiltrates in only 5 of the 12 sIBM patients tested (among these 5 patients, 2.5±1.6% of CD4^+^ cells were positive for IL-17, Figure S1). In muscle lysates from sIBM patients, only CXCL-10 levels were significantly increased compared to normal muscle (2.3±2.8 pg/mg *vs.* 0.15±0.05 pg/mg, p = 0.02), while IL-1RA and CCL-2 were increased only in some sIBM patients (data not shown).

**Figure 2:**
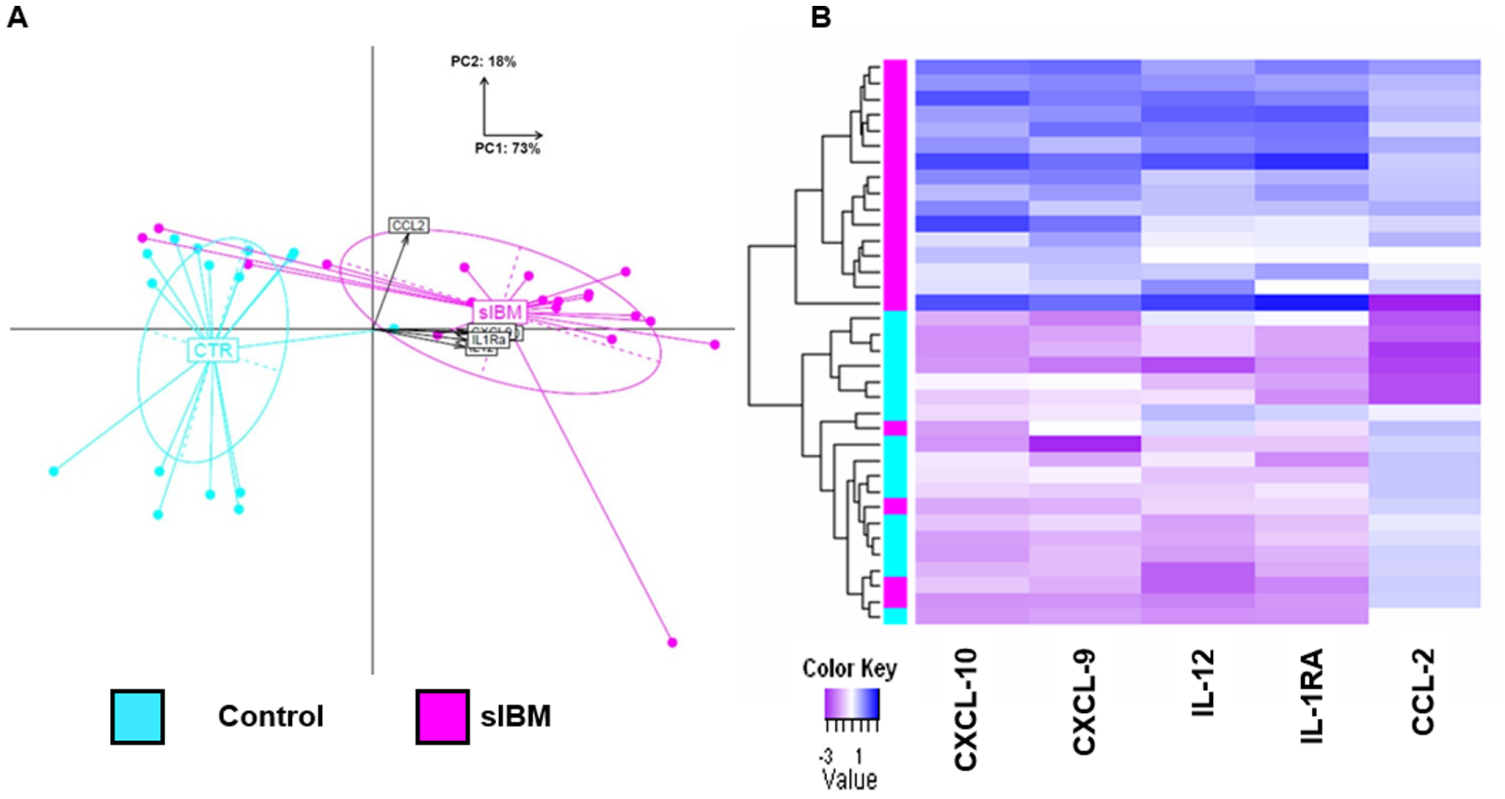
Unsupervised analyses using the Th1 signature. **A.** Principal Components Analysis (PCA) based on the selected Th1 signature. The projection of each individual on the first two PCA components using the five cytokine expression levels identified as significantly increased in sIBM patients (p<0.05) revealed that sIBM patients (magenta) are distant from controls (cyan). **B.** Hierarchical clustering (Euclidean distance; complete linkage method) based on the expression pattern of the five cytokines across the sIBM patients (magenta) and controls.

The five serum markers increased in sIBM patients included mostly Th1 cytokines and chemokines: CXCL-9, CXCL-10, IL-12, CCL-2, and IL-1RA. These five markers allowed a good segregation of sIBM patients from healthy age-matched controls (Figure 2. A–B), enabling us to build a predictive model with an accuracy of 83%.

In another set of analysis including 9 sIBM patients (different from those included in the first set) we compared cytokines results to other myositis controls (DM, PM, and SRP IMNM patients). Significant variation of cytokine levels in sera such as IL-1RA or CCL-2 (Table 1) was observed but did not allow segregating sIBM patients from others myositis controls because of overlap between groups (Figure S2).

**Table 1:** Cytokine and chemokine serum level in sIBM patients and myositis controls.

	sIbm	DM	PM	IMNM
**IFN-α**	21.1±8.2	33.9±15.2*	34.3±16.2*	33.4±23.2*
**IFN-γ**	1.3±0.9	1.2±1.2	1.1±1.1	0.8±0.5
**IL-1-RA**	208.6±145.8	140±68.1	181.6±154.5	128.8±92.1*
**IL-1b**	17.3±27.9	34.2±95.6	10.8±19.4	18.5±41.3
**IL-2**	5.1±9.3	9.9±29.4	2.7±6.3	11.6±36.4
**Il-2R**	223.9±105.6	333.9±189.1	516.9±606.3	228.9±78.6
**IL-4**	6.9±4.1	6.7±1.2	6.8±2.4	7.7±2
**IL-5**	0.1±1.7	0.4±0.3	0.4±0.3	0.5±0.3
**IL-6**	8.1±5.1	6.2±7.4	9.3±10.5	4.1±2.4
**IL-7**	8.0±2.5	3.9±6.0	7.9±12.1	6.3±7.8
**IL-8**	24.6±18.3	75.2±143.5	41.8±39.9	682.8±2763
**IL-10**	2.7±2.3	2.2±2.1	4.0±7.1	6.8±14.1
**IL-12**	211.8±89	173.4±44.6	266.9±261.8	152.3±56.8
**IL-13**	2.5±1.1	1.9±1.4	1.4±1	1.1±0.9
**IL-15**	55.7±59.7	93.9±54.7*	111±109.8	21.6±7.3
**IL-17**	12.4±22.4	14.5±14.5	8.7±10.1	16.6±14.6
**CXCL-10**	84.6±93.9	219.3±131.5*	202.8±201.9	37.4±16.4
**CXCL-9**	56.3±75.4	556.7±1033.0	27.7±34.9	11.3±7.0
**CCL-2**	793.1±329.8	1493.0±403.4*	2018±2303*	1101±557.8
**CCL-3**	57.8±78.4	30.2±18.8	49.7±74.7	100.2±172.3
**CCL-4**	78.1±44.5	97.8±88.0	120.5±111.1	158.9±200.9
**CCL-5**	18608±2051	19752±1491	17654±4342	18992±1665
**CCL-11**	77.6±59.9	90.3±45.4	64.5±33.73	57.4±17.8
**G-CSF**	11.2±13.1	4.4±0.9*	7.5±10.4*	13.7±15.3
**TNF-α**	2.9±2.4	2.8±1.3	2.4±1.1	5.1±5.6

Mean serum levels (±SD) of cytokines and chemokines are represented (pg/mL) for sIBM patients and DM, PM, IMNM patients. (*) shows significant difference (p<0.05) compared to sIBM patients.

### Th-1 Lineage is Associated with an Increased Frequency of Circulating Late Effector Memory T Cells in sIBM Patients

In an attempt to define the phenotype of the cells involved in the immune response engaged in a Th-1 lineage, FACS analysis were performed on fresh PBMCs from sIBM and matched healthy controls (age-matched to account for immunosenescence [11], [12]).

**Figure 3:**
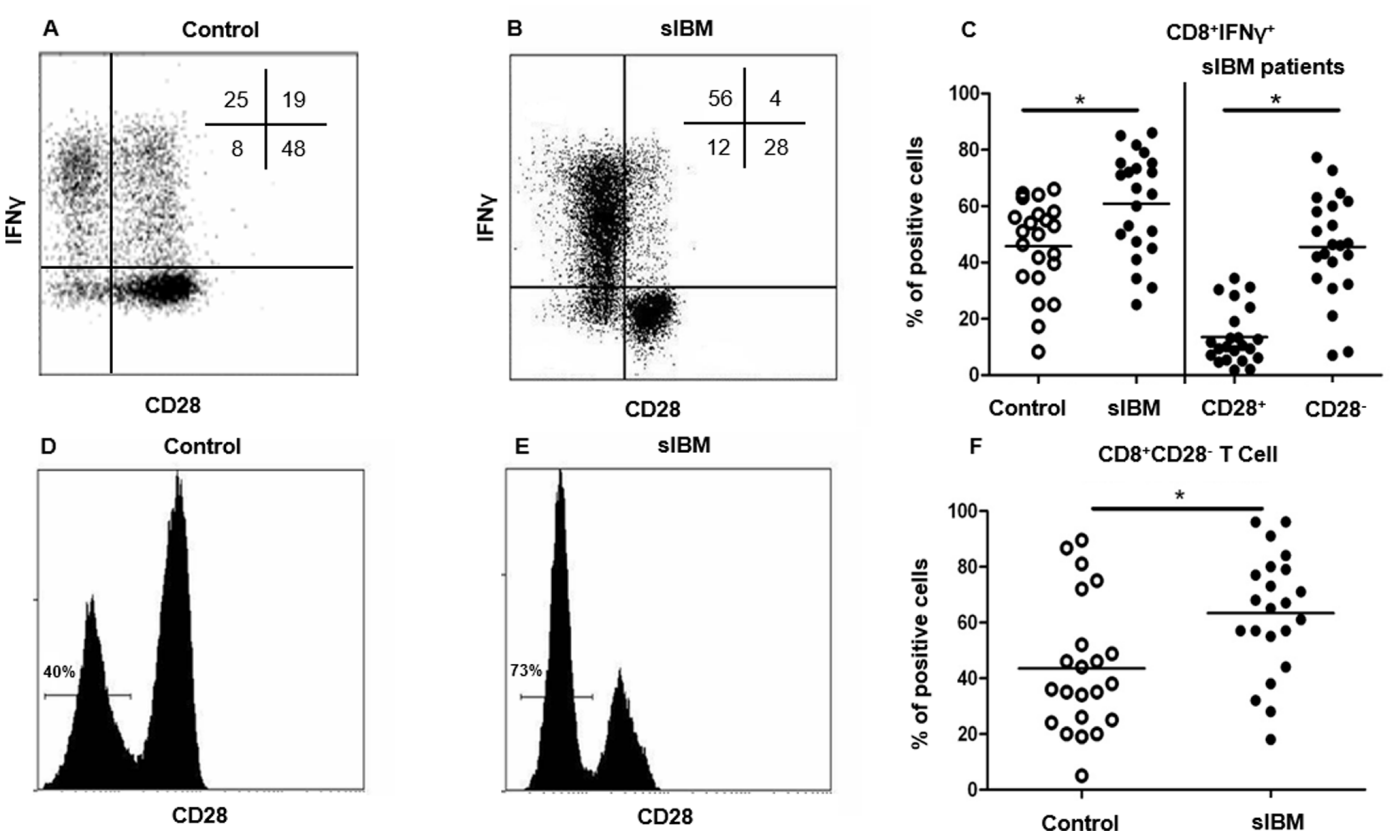
Interferon γ production by T cells in sIBM patients depending on CD28 and naive/memory phenotype. Representative flow cytometry analysis showing IFN-γ production in CD28^−^ and CD28^+^CD8^+^ T cells in one control (**A**) and one sIBM patient (**B**). The percentage of each subpopulation is shown in the upper right corner. Pooled data showing IFN-γ^+^CD8^+^ T cells from sIBM patients and controls (p = 0.01) (left side). Pooled data showing IFN-γ+ cells among CD28^+^ and CD28-CD8^+^ T cells, represented as percentage (p<0.0001) (right side) (**C**). Representative flow cytometry analysis showing CD28^−^ and CD28^+^CD8^+^ T cell subpopulations in one representative control (**D**) and sIBM patient (**E**). Pooled data showing CD28^−^ CD8^+^ T cells from IBM patients and controls (p = 0.001) (**F**). Horizontal lines indicate means. *p<0.05.

While the percentage of CD4^+^IFNγ^+^ T cells in peripheral compartment was not significantly different between sIBM patients and controls (12.9±6% *vs.* 10.8±5.8%; p = 0.7), the percentage of CD8^+^IFNγ^+^ T cells was higher in sIBM patients than in controls (60.8±18.0% *vs.* 45.8±16.0%; p = 0.01, Figure 3. A–C). IFNγ^+^ cells were mainly CD8^+^CD28^−^ T cells, since of 45.6±18.5% CD8^+^CD28^−^ T cells were IFNγ^+^ whereas only 13.5±9.9% CD8^+^CD28^+^ T cells were IFNγ^+^ (p<0.0001, Figure 3. A–C). This result is in agreement with the increased percentage of CD8^+^CD28^−^ T cells in sIBM patients compared with controls (63.2±12.5% *vs.* 43.5±23.5%; p = 0.001, Figure 3. D–F).

Knowing that CD8^+^CD28^−^ T cells have a memory phenotype [13], we wanted to define the naïve/memory ratio of T cells, based on CD45RA and CD62L expression. We defined naïve (Naive: CD45RA^+^CD62L^+^), central memory (CM: CD45RA^−^CD62L^+^), early effector memory (EM: CD45RA^−^CD62L^−^) and late effector memory (LM: CD45RA^+^CD62L^−^) T cells.

**Figure 4:**
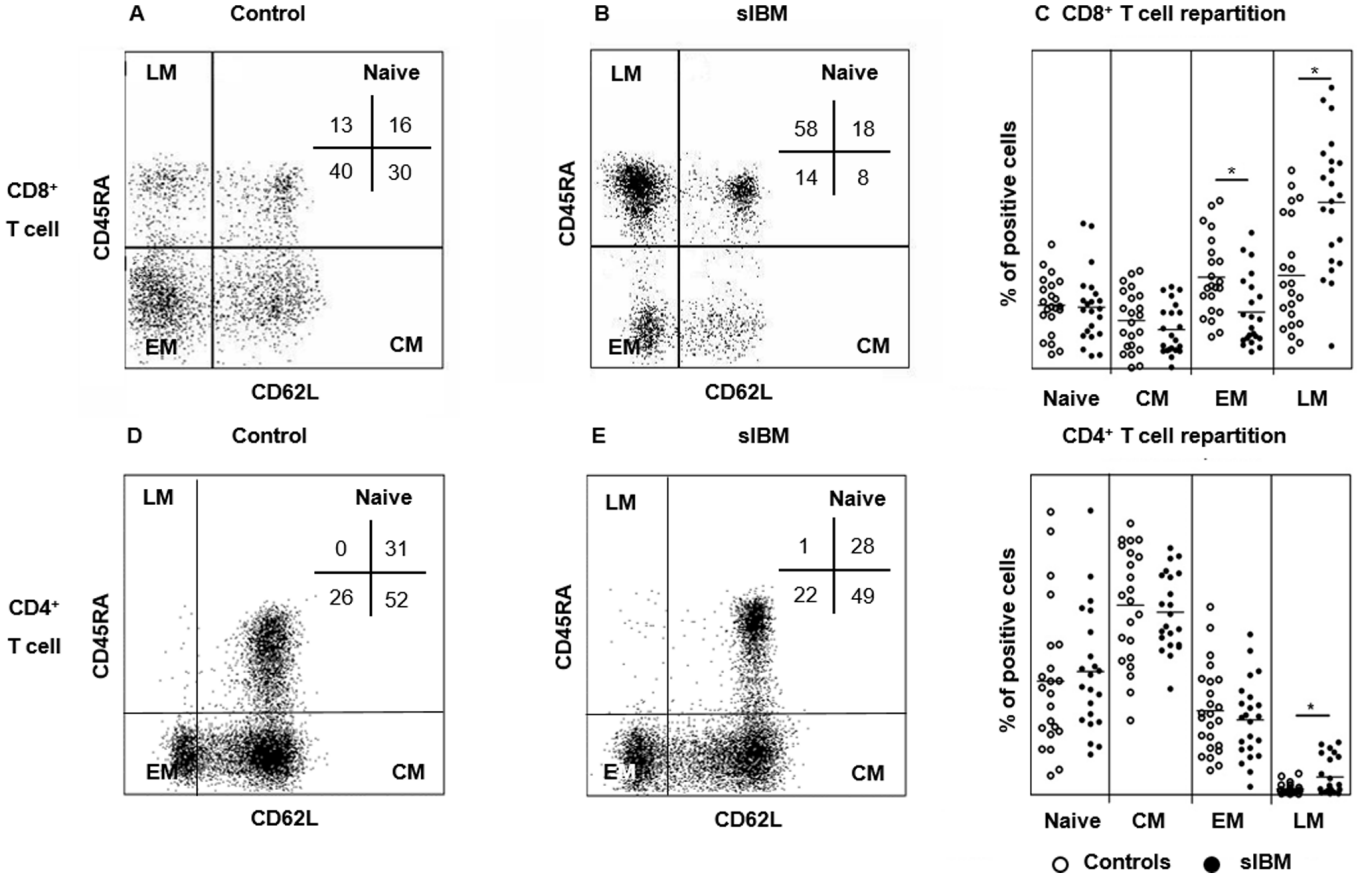
Naive/memory phenotype T cell repartition in sIBM patients. Representative flow cytometry analysis showing the gating strategy used to define naive T cells (Naïve: CD45RA^+^CD62L^+^), central memory T cells (CM: CD45RA^−^CD62L^+^), early activated memory T cells (EM: CD45RA^−^CD62L^−^) and late effector memory T cells (LM: CD45RA^+^CD62L^−^) subsets of CD8^+^ T cells, in one representative healthy control (**A**) and sIBM patient (**B**). The percentage of each subpopulation among CD8^+^ T cells is represented in the upper right quadrant. Pooled data showing naive and memory CD8^+^ T cell subpopulations from sIBM patients and controls (p = 0.004 and p = 0.003 for LM and EM respectively) (**C**). The same strategy was used for the CD4^+^ T cell compartment. Representative flow cytometry analysis of CD4^+^ T cells showing the gating strategy in one representative healthy control (**D**) and one representative sIBM patient (**E**). Pooled data showing naive and memory subpopulations of CD4^+^ T cells from IBM patients and controls (p = 0.003 for LM) (**F**). Horizontal lines indicate means. *p<0.05.

In the CD8^+^ T cell compartment (Figure 4. A–C), sIBM patients had a higher percentage of LM compared to controls (52.2±20.7% *vs.* 29.2±17.5%, p<0.005) and a lower percentage of EM (17.7±11.0% *vs*. 28.7±12.4%, p = 0.003, Figure 4. A–C). The percentage of CD4^+^ LM was higher in the sIBM patients (4.6±4.6% *vs.* 1.5±1.3%; p = 0.04, Figure 4. D–F) whereas the percentage of CD4^+^ Naive, CM, and EM were similar (Figure 4. D–F).

### sIBM Patients Exhibit Decreased Frequency of Circulating Tregs with Normal Suppressive Functions

**Figure 5:**
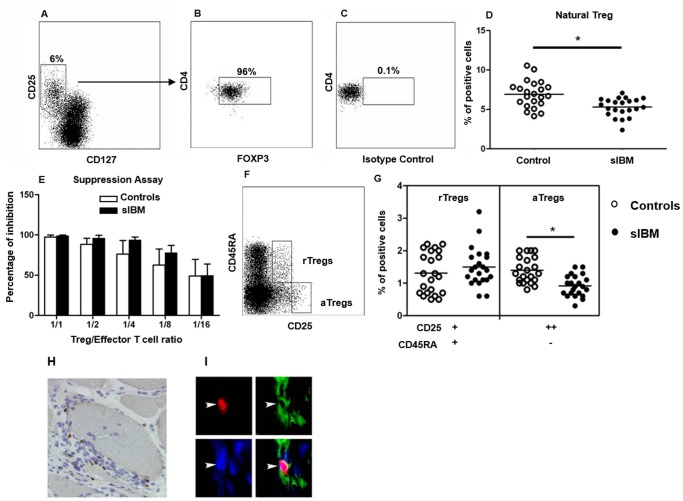
Analysis of Tregs in sIBM patients. Representative flow cytometry analysis of CD25^+^CD127^low^ subpopulations among CD3^+^CD4^+^ T lymphocytes in a control subject (**A**). Tregs were defined as Foxp3+ cells among the previously gated CD3^+^CD4^+^CD25^+^CD127^low^ subpopulation (**B**). Non-specific staining (isotype control) was used to define FoxP3^+^ cells (**C**). Pooled data showing the percentage of Tregs among CD4^+^ T cells from sIBM patients and controls in the systemic compartment (p = 0.01). Horizontal lines indicate means (**D**). Percentage of inhibition (±SD; n = 4) of Tregs on the proliferation of autologous CD4^+^CD25^−^ T cells (responders). Responder+allogeneic MNCs were used as positive control. Proliferation of responder cells was measured by 3H-Tdr incorporation (counts per minute, cpm) (**E**). Representative flow cytometry analysis showing resting Tregs (rTregs: CD45RA^+^CD25^+^) and activated Tregs (aTregs: CD45RA^−^CD25^high^) among CD4^+^ T lymphocytes in a control subject (**F**). Pooled data showing rTregs and aTregs from sIBM patients and controls (p = 0.0003) (**G**). Immunostaining of a muscle section from a sIBM patient, showing Foxp3^+^ cells (**H**) in the muscle infiltrate. Immunofluorescence staining showing Foxp3^+^ cells (red) within the muscle infiltrate in a sIBM patients (**I**). The upper left corner shows a FoxP3^+^ cell (red), the upper right corner shows CD4^+^ cells (green), the lower left corner shows nuclear staining (dapi); coexpression of CD4 and FoxP3 is shown by merged fluorescence images in the lower right corner. Horizontal lines indicate means; *p<0.05.

We tested Tregs, which are involved in the maintenance of peripheral tolerance [14]. We observed a lower percentage of natural Tregs (CD4^+^CD25^+^CD127^low^FOXP3^+^ T cells, Figure 5. A–D) in sIBM patients compared to controls (5.2±1.1% *vs.* 6.9±1.7%; p = 0.01, Figure 5. D). The functional suppressive effect of Tregs from sIBM patients on the proliferation of autologous T cells was similar to that observed in controls (Figure 5. E, n = 4).

Because FoxP3 can be transiently expressed by activated CD4^+^ T cells [15], we analysed CD45RA expression (Figure 5) to distinguish activated conventional T cells (CD3^+^CD4^+^CD25^+^CD45RA^−^, that are Foxp3^+^) from activated Tregs (aTregs: CD3^+^CD4^+^CD25^high^CD45RA^−^, that are Foxp3^high^, and from resting Tregs (rTregs: CD3^+^CD4^+^ CD25^high^CD45RA^+^, that are Foxp3^+^) [16] (Figure 5. F–G). We observed a lower percentage of aTregs among CD4^+^ T cells in sIBM patients than in controls (0.9±0.3% *vs.* 1.4±0.4%; p = 0.0003, Figure 5), whereas no statistically significant difference was found in rTregs (Figure 5).

We also examined the presence of Foxp3^+^ cells in sIBM muscle sections (Figure 5. H). These cells represented 18.7±7.4% of all CD4^+^ cells. Co-expression of FoxP3 and CD4 by cells infiltrating muscle was confirmed with double immunofluorescence staining (Figure 5. I).

## Discussion

The aetiology of sIBM, is poorly understood and the role of the immune system in the disease pathophysiology is not completely characterized.

In this study, we show that sIBM patients have increased levels of Th1 chemokines and cytokines (CXCL-9, CXCL-10 and IL-12) associated with an increased level of CD8^+^CD28^−^ T cells prone to produce large amounts of IFN-γ. Moreover, Tregs are found within sIBM muscle infiltrates, and the percentage of peripheral Tregs is lower than in age-matched controls. Treg cells from sIBM patients were functional and remained able to suppress effector T cells.

While these findings reveal an ongoing immune response with a Th-1 lineage in sIBM patients, the apparent unbalance in the frequency of Tregs in peripheral blood may represent a feature of sIBM as it was described for other autoimmune disorders [14].

Conversely, in this study we did not observe Th17 polarization in sIBM patients, neither in blood nor in muscle biopsies, arguing against a possible pathophysiological role of Th17 cells in the disease. We and others reported increased serum levels of Th-1 chemokines CXCL-9 and CXCL-10 [17], which is in accordance with previous data reporting the presence of these chemokines in muscle of sIBM [18]–[19], associated with strong CXCR-3 expression in the majority of muscle-infiltrating T cells [19]. Th1 immune responses permitting CXCR3-mediated CD8^+^ memory T cells migration to inflamed tissues [17] is driven by CXCL-9 and CXCL-10, that are two IFN-γ-inducible chemokines.

This overexpression of Th1 cytokines and chemokines is not specific of sIBM since, unlike previous studies [20], it does not permit to delineate disease-specific profiles and identify diagnostic patterns allowing the discrimination of sIBM from the other myositis.

We also observed a significant increase in the production of IFN-γ by CD8^+^ T cells in sIBM patients. We characterized these cells as CD8^+^CD28^−^ T cells and as having a memory phenotype. Further analyses allowed us to detect a significant percentage of CD8^+^CD28^−^ T cells corresponding to the increased percentage of CD8^+^ LM since the loss of CD28 is associated with antigen recognition [13]. These results are in agreement with previous observations in sIBM patients showing an increased frequency of oligoclonal CD8^+^CD28^−^ T cells in the periphery with higher degranulation potential associated with an accumulation of clonally expanded CD8^+^CD28^−^ T cells within muscle infiltrates [2], [21].

Here we also looked at the Treg compartment in our cohort of sIBM subjects. The absence of Treg due to mutations in the Foxp3 gene, which encodes for a key transcription factor governing Treg differentiation, causes a multiorgan autoimmune disease in humans known as IPEX syndrome [22]. Tregs play a key role in the control in peripheral immune tolerance [23]. To our knowledge, the Treg deficiency we observed in the blood of sIBM patients had never been reported, while a decrease in frequency of peripheral Tregs has been described in other forms of myositis, including DM and PM [24], [25], as well as in other autoimmune diseases [26]–[28]. Although Foxp3 can be expressed by activated T cells that do not display suppressive functions [15], we confirmed, based on CD45RA expression [16], that FoxP3^+^ cells correspond to true Tregs and that the observed Tregs decrease concerned mainly aTregs. We also showed for the first time that Tregs from sIBM patients display normal function. This is suggestive of a systemic Treg deficiency, which could be due to a global quantitative Treg deficiency or to a Treg migration from the periphery to inflamed muscle, since we detected Foxp3^+^ cells in large number in sIBM muscle biopsies.

As activated T cells can be converted into Tregs in specific conditions [29], muscle-infiltrating Tregs could also correspond to induce Tregs.

As stated above, some Foxp3^+^ cells may correspond to activated T cells, but in this case Foxp3 expression would be below the detection limit of immunohistological methods [30]. We observed no functional deficit of circulating Tregs in sIBM patients, whereas impaired function has been observed in other autoimmune diseases such as multiple sclerosis [31]. A functional deficit of muscle-infiltrating Tregs cannot be excluded, but the number of cells that can be extracted from muscle is too low to perform *ex vivo* functional analyses.

In conclusion, in our study the analysis of serum cytokines and the immunophenotyping in peripheral blood and muscle in sIBM patients, highlights a systemic immune activation with Th1 polarization involving IFN-γ pathway and CD8^+^CD28^−^T cells associated with peripheral Tregs cell deficiency. We were not able to show to what extent this immune activation is specific to sIBM compared to other myositis. Nevertheless therapeutic approaches targeting effector Th1 T cells and/or sparing or increasing Treg numbers might be of interest in the treatment of sIBM.

## Supporting Information

Figure S1 **Percentage of CD4^+^IL-17^+^ T cells in sIBM.** Pooled data showing serum levels (pg/ml) of IL-17 in sIBM patients (open circles) and controls (full circles) (**A**). Representative flow cytometry showing gate strategy to define IL-17^+^CCR6^+^CD4^+^ T cells subpopulations in one sIBM patient (**B**). Pooled data showing percentage of IL-17^+^CCR6^+^ among CD4+ T cells from sIBM patients and controls in the systemic compartment (**C**). Immunostaining of a representative muscle section from a sIBM patient showing no IL-17 positive cells (**D**) among infiltrate containing CD4^+^ T cells (**E**). Immunostaining of a muscle section from a sIBM showing an IL-17^+^ cell (**F**).
(TIF)

Figure S2 **Cytokines and chemokines in sIBM patients compare to myositis controls. A.** Radar chart representing the 25 chemokines and cytokines for sIBM, DM, PM and IMNM patients. Non myositis control (blue dot line) is also represented. Values of each cytokines are expressed in pg/mL. **B.** Hierarchical clustering (Euclidean distance; complete linkage method) based on the expression pattern of the six cytokines significant in sIBM compare to myositis controls (G-CSF, IFN-α, IL-15, IL-1RA, CXCL-10 and CCL-2) across myositides patients including: sIBM patients, DM patients, PM patients and IMNM patients, do not permitted to segregated different groups. **C.** Projection by principal component analysis (PCA) of sIBM, DM, PM and IMNM patients using the expression levels of IFN-α, CCL-2 and CXCL10 according to the first two principal components (PC). PC1 and PC2 capture respectively 72% and 14% of the total variability. The three cytokines are highly correlated and do not allow to distinguish the four pathologies.
(TIF)

Table S1 **Patients’ characteristics.**
(DOCX)

## References

[1] Benveniste O, Guiguet M, Freebody J, Dubourg O, Squier W, et al (2011) Long-term observational study of sporadic body inclusion body myositis. Brain 134(Pt11): 3176–84. 21994327 10.1093/brain/awr213

[2] Dimitri D, Benveniste O, Dubourg O, Maisonobe T, Eymard B, et al (2006) Shared blood and muscle CD8+ T-cell expansions in inclusion body myositis. Brain, 129(Pt 4): 986–995. 10.1093/brain/awl020 16455793

[3] Askanas V, Engel WK (2008) Inclusion-body myositis: muscle-fiber molecular pathology and possible pathogenic significance of its similarity to Alzheimer’s and Parkinson’s disease brains. Acta Neuropathol 116(6): 583–595. 18974994 10.1007/s00401-008-0449-0 PMC2635944

[4] Greenberg SA (2010) Comment on alemtuzumab and inclusion body myositis. Brain, 133(Pt 5): e135; author reply e136. 10.1093/brain/awp276 PMC2859147 19892769

[5] Griggs RC, Askanas V, DiMauro S, Engel A, Karpati G, et al (1995) Inclusion body myositis and myopathies. Ann Neurol 38(5): 705–713. 7486861 10.1002/ana.410380504

[6] Dubourg O, Wanschitz J, Maisonobe T, Behin A, Allenbach Y, et al (2011) Diagnostic value of markers of muscle degeneration in sporadic inclusion body myositis. Acta Myol 30(2): 103–108. 22106712 PMC3235833

[7] Hoogendijk JE, Amato AA, Lecky BR, Choy EH, Lundberg IE, et al (2004) 119th ENMC international workshop: trial design in adult idiopathic inflammatory myopathies, with the exception of inclusion body myositis, 10–12 October 2003, Naarden, The Netherlands. Neuromuscul Disord 14(5): 337–345. 15099594 10.1016/j.nmd.2004.02.006

[8] Guillot-Delost M, Cherai M, Hamel Y, Rosenzwajg M, Baillou C, et al (2008) Clinical-grade preparation of human natural regulatory T-cells encoding the thymidine kinase suicide gene as a safety gene. J Gene Med 10(8): 834–846. 18615770 10.1002/jgm.1220

[9] Baird GS, Montine TJ (2008) Multiplex immunoassay analysis of cytokines in idiopathic inflammatory myopathy. Arch Pathol Lab Med 132(2): 232–238. 18251582 10.5858/2008-132-232-MIAOCI

[10] Terrier B, Geri G, Chaara W, Allenbach Y, Rosenzwajg M, et al (2012) Interleukin-21 modulates Th1 and Th17 responses in giant cell arteritis. Arthritis Rheum 64(6): 2001–2011. 22147555 10.1002/art.34327

[11] Effros RB, Dagarag M, Spaulding C, Man J (2005) The role of CD8+ T-cell replicative senescence in human aging. Immunol Rev 205: 147–157. 15882351 10.1111/j.0105-2896.2005.00259.x

[12] Gregg R, Smith CM, Clark FJ, Dunnion D, Khan N, et al (2005) The number of human peripheral blood CD4+ CD25high regulatory T cells increases with age. Clin Exp Immunol 140(3): 540–546. 15932517 10.1111/j.1365-2249.2005.02798.x PMC1809384

[13] Appay V, van Lier RA, Sallusto F, Roederer M (2008) Phenotype and function of human T lymphocyte subsets: consensus and issues. Cytometry A 73(11): 975–983. 18785267 10.1002/cyto.a.20643

[14] Sakaguchi S, Yamaguchi T, Nomura T, Ono M (2008) Regulatory T cells and immune tolerance. Cell 133(5): 775–787. 18510923 10.1016/j.cell.2008.05.009

[15] Wang J, Ioan-Facsinay A, van der Voort EI, Huizinga TW, Toes RE (2007) Transient expression of FOXP3 in human activated nonregulatory CD4+ T cells. Eur J Immunol 37(1): 129–138. 17154262 10.1002/eji.200636435

[16] Miyara M, Yoshioka Y, Kitoh A, Shima T, Wing K, et al (2009) Functional delineation and differentiation dynamics of human CD4+ T cells expressing the FoxP3 transcription factor. Immunity 30(6): 899–911. 19464196 10.1016/j.immuni.2009.03.019

[17] Lacotte S, Brun S, Muller S, Dumortier H (2009) CXCR3, inflammation, and autoimmune diseases. Ann N Y Acad Sci 1173: 310–317. 19758167 10.1111/j.1749-6632.2009.04813.x

[18] Schmidt J, Barthel K, Wrede A, Salajegheh M, Bahr M, et al (2008) Interrelation of inflammation and APP in sIBM: IL-1 beta induces accumulation of beta-amyloid in skeletal muscle. Brain, 131(Pt 5): 1228–1240. 10.1093/brain/awn053 PMC2367696 18420712

[19] De Paepe B, De Keyzer K, Martin JJ, De Bleecker JL (2005) Alpha-chemokine receptors CXCR1–3 and their ligands in idiopathic inflammatory myopathies. Acta Neuropathol 109(6): 576–582. 15937690 10.1007/s00401-005-0989-5

[20] Szodoray P, Alex P, Knowlton N, Centola M, Dozmorov I, et al (2010) Idiopathic inflammatory myopathies, signified by distinctive peripheral cytokines, chemokines and the TNF family members B-cell activating factor and a proliferation inducing ligand. Rheumatology (Oxford 49(10): 1867–1877. 20591831 10.1093/rheumatology/keq151 PMC2936946

[21] Pandya JM, Fasth AE, Zong M, Arnardottir S, Dani L, et al (2010) Expanded T cell receptor Vbeta-restricted T cells from patients with sporadic inclusion body myositis are proinflammatory and cytotoxic CD28null T cells. Arthritis Rheum 62(11): 3457–3466. 20662057 10.1002/art.27665

[22] Bennett CL, Christie J, Ramsdell F, Brunkow ME, Ferguson PJ, et al (2001) The immune dysregulation, polyendocrinopathy, enteropathy, X-linked syndrome (IPEX) is caused by mutations of FOXP3. Nat Genet 27(1): 20–21. 11137993 10.1038/83713

[23] Tang Q, Bluestone JA (2008) The Foxp3+ regulatory T cell: a jack of all trades, master of regulation. Nat Immunol 9(3): 239–244. 18285775 10.1038/ni1572 PMC3075612

[24] Banica L, Besliu A, Pistol G, Stavaru C, Ionescu R, et al (2009) Quantification and molecular characterization of regulatory T cells in connective tissue diseases. Autoimmunity 42(1): 41–49. 18800250 10.1080/08916930802282651

[25] Antiga E, Kretz CC, Klembt R, Massi D, Ruland V, et al (2010) Characterization of regulatory T cells in patients with dermatomyositis. J Autoimmun 35(4): 342–350. 20843660 10.1016/j.jaut.2010.07.006

[26] Chi LJ, Wang HB, Zhang Y, Wang WZ (2007) Abnormality of circulating CD4(+)CD25(+) regulatory T cell in patients with Guillain-Barre syndrome. J Neuroimmunol 192(1–2): 206–214. 17997492 10.1016/j.jneuroim.2007.09.034

[27] Zhang B, Zhang X, Tang F, Zhu L, Liu Y (2008) Reduction of forkhead box P3 levels in CD4+CD25high T cells in patients with new-onset systemic lupus erythematosus. Clin Exp Immunol 153(2): 182–187. 18505426 10.1111/j.1365-2249.2008.03686.x PMC2492897

[28] Li X, Xiao BG, Xi JY, Lu CZ, Lu JH (2008) Decrease of CD4(+)CD25(high)Foxp3(+) regulatory T cells and elevation of CD19(+)BAFF-R(+) B cells and soluble ICAM-1 in myasthenia gravis. Clin Immunol 126(2): 180–188. 18054287 10.1016/j.clim.2007.10.001

[29] Chen W, Jin W, Hardegen N, Lei KJ, Li L, et al (2003) Conversion of peripheral CD4+CD25− naive T cells to CD4+CD25+ regulatory T cells by TGF-beta induction of transcription factor Foxp3. J Exp Med 198(12): 1875–1886. 14676299 10.1084/jem.20030152 PMC2194145

[30] Taflin C, Miyara M, Nochy D, Valeyre D, Naccache JM, et al (2009) FoxP3+ regulatory T cells suppress early stages of granuloma formation but have little impact on sarcoidosis lesions. Am J Pathol 174(2): 497–508. 19147826 10.2353/ajpath.2009.080580 PMC2630558

[31] Viglietta V, Baecher-Allan C, Weiner HL, Hafler DA (2004) Loss of functional suppression by CD4+CD25+ regulatory T cells in patients with multiple sclerosis. J Exp Med 199(7): 971–979. 15067033 10.1084/jem.20031579 PMC2211881

