# Supplementary figures and images for: Th1 Response and Systemic Treg Deficiency in Inclusion Body Myositis
Source: PLoS One. 2014 Mar 4;9(3):e88788. doi: 10.1371/journal.pone.0088788 (PMC3942319; doi:10.1371/journal.pone.0088788)

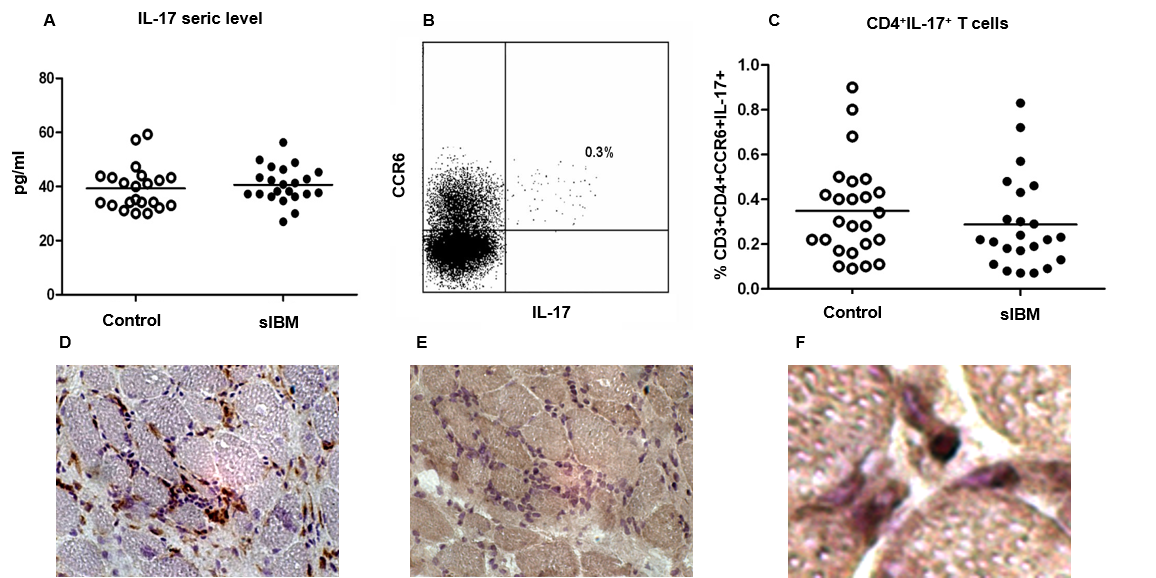

Supplement: Figure S1 — Percentage of CD4+IL-17+ T cells in sIBM. Pooled data showing serum levels (pg/ml) of IL-17 in sIBM patients (open circles) and controls (full circles) (A). Representative flow cytometry showing gate strategy to define IL-17+CCR6+CD4+ T cells subpopulations in one sIBM patient (B). Pooled data showing percentage of IL-17+CCR6+ among CD4+ T cells from sIBM patients and controls in the systemic compartment (C). Immunostaining of a representative muscle section from a sIBM patient showing no IL-17 positive cells (D) among infiltrate containing CD4+ T cells (E). Immunostaining of a muscle section from a sIBM showing an IL-17+ cell (F). (TIF) [file pone.0088788.s001.tif]

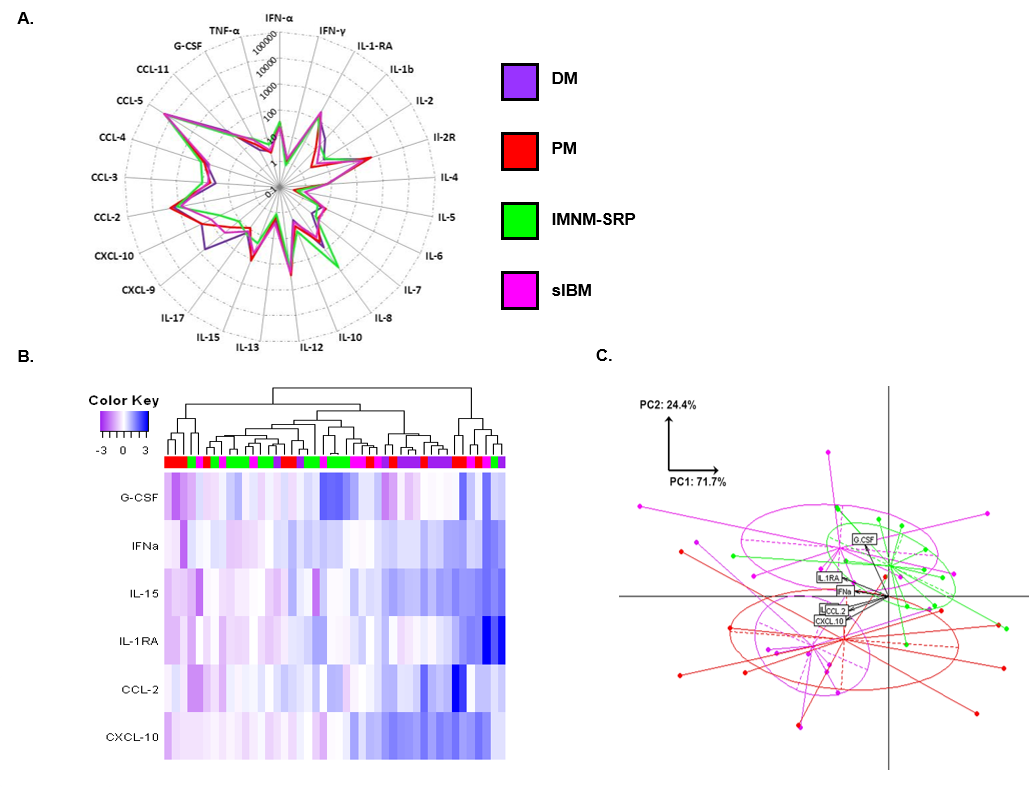

Supplement: Figure S2 — Cytokines and chemokines in sIBM patients compare to myositis controls. A. Radar chart representing the 25 chemokines and cytokines for sIBM, DM, PM and IMNM patients. Non myositis control (blue dot line) is also represented. Values of each cytokines are expressed in pg/mL. B. Hierarchical clustering (Euclidean distance; complete linkage method) based on the expression pattern of the six cytokines significant in sIBM compare to myositis controls (G-CSF, IFN-α, IL-15, IL-1RA, CXCL-10 and CCL-2) across myositides patients including: sIBM patients, DM patients, PM patients and IMNM patients, do not permitted to segregated different groups. C. Projection by principal component analysis (PCA) of sIBM, DM, PM and IMNM patients using the expression levels of IFN-α, CCL-2 and CXCL10 according to the first two principal components (PC). PC1 and PC2 capture respectively 72% and 14% of the total variability. The three cytokines are highly correlated and do not allow to distinguish the four pathologies. (TIF) [file pone.0088788.s002.tif]
