# Supplementary material for: Th1 Response and Systemic Treg Deficiency in Inclusion Body Myositis
Source: PLoS One. 2014 Mar 4;9(3):e88788. doi: 10.1371/journal.pone.0088788 (PMC3942319; doi:10.1371/journal.pone.0088788)
Supplement: Table S1 — Patients’ characteristics. (DOCX) [file pone.0088788.s003.docx]

**Table S1. Patients’ characteristics**
